# Regional QT Interval Dispersion as an Early Predictor of Reperfusion in Patients with Acute Myocardial Infarction after Fibrinolytic Therapy

**DOI:** 10.5935/abc.20180239

**Published:** 2019-01

**Authors:** Gabriel Dotta, Francisco Antonio Helfenstein Fonseca, Maria Cristina de Oliveira Izar, Marco Tulio de Souza, Flavio Tocci Moreira, Luiz Fernando Muniz Pinheiro, Adriano Henrique Pereira Barbosa, Adriano Mendes Caixeta, Rui Manoel Santos Póvoa, Antônio Carlos Carvalho, Henrique Tria Bianco

**Affiliations:** Universidade Federal de São Paulo, São Paulo, SP - Brazil

**Keywords:** ST Elevation Myocardial Infarction, Electrocardiography, Myocardial Reperfusion, Percutaneous Coronary Intervention, Biomarkers

## Abstract

**Background:**

Patients with ST-elevation acute myocardial infarction attending primary care centers, treated with pharmaco-invasive strategy, are submitted to coronary angiography within 2-24 hours of fibrinolytic treatment. In this context, the knowledge about biomarkers of reperfusion, such as 50% ST-segment resolution is crucial.

**Objective:**

To evaluate the performance of QT interval dispersion in addition to other classical criteria, as an early marker of reperfusion after thrombolytic therapy.

**Methods:**

Observational study including 104 patients treated with tenecteplase (TNK), referred for a tertiary hospital. Electrocardiographic analysis consisted of measurements of the QT interval and QT dispersion in the 12 leads or in the ST-segment elevation area prior to and 60 minutes after TNK administration. All patients underwent angiography, with determination of TIMI flow and Blush grade in the culprit artery. P-values < 0.05 were considered statistically significant.

**Results:**

We found an increase in regional dispersion of the QT interval, corrected for heart rate (regional QTcD) 60 minutes after thrombolysis (p = 0.06) in anterior wall infarction in patients with TIMI flow 3 and Blush grade 3 [T3B3(+)]. When regional QTcD was added to the electrocardiographic criteria for reperfusion (i.e., > 50% ST-segment resolution), the area under the curve increased to 0.87 [(0.78-0.96). 95% IC. p < 0.001] in patients with coronary flow of T3B3(+). In patients with ST-segment resolution >50% and regional QTcD > 13 ms, we found a 93% sensitivity and 71% specificity for reperfusion in T3B3(+), and 6% of patients with successful reperfusion were reclassified.

**Conclusion:**

Our data suggest that regional QTcD is a promising non-invasive instrument for detection of reperfusion in the culprit artery 60 minutes after thrombolysis.

## Introduction

Despite advances in its treatment, acute myocardial infarction (AMI) rates are still high. In this regard, reperfusion of the culprit artery has become the main objective of ST-elevation acute myocardial infarction (STEMI) treatment. Early reperfusion with preservation of arterial permeability is responsible for mortality reduction in the acute phase, and in medium and long term.^1^^,^^2^ Nevertheless, once arterial flow is reestablished, myocardial stunning is not resolved due to the injury-reperfusion process.^3^^,^^4^

Primary percutaneous coronary intervention (PCI) is considered the gold standard for the treatment of STEMI.^5^ Nevertheless, when PCI is not available or cannot be performed in a timely manner, pharmaco-invasive strategy (PIS) is an alternative for reperfusion, consisted of intravenous fibrinolysis, conducted in primary or prehospital care.^6^^-^^8^ Classical criteria for reperfusion include improvement of ischemic symptoms and ST-segment resolution (> 50% in the highest lead within 60-90 min of fibrinolytic administration).^9^^,^^10^

There is some controversy about the behavior of the heart rate-corrected QT interval (QTc) after STEMI. While some studies have reported an increase in QTc in the acute phase followed by its decrease after reperfusion, others reported increased QTc, which was associated with non-reperfusion.^11^^,^^12^ QTc dispersion (QTcD) was reduced in patients with successful fibrinolytic therapy and decreased in non-revascularized patients. A reduction in QTcD after fibrinolysis was predictive of coronary reperfusion.^13^ There is evidence that recanalization after an acute event is associated with a decrease in QTcD, as observed in the TEAM-2 and TEAM-3 studies.^14^^,^^15^ To our knowledge, there is no study on QTcD after PIS combined with angiographic perfusion imaging (TIMI flow and Blush grade) after tenecteplase (TNK) administration.

Therefore, our study aimed to evaluate the behavior of QTcD in electrocardiography (ECG) before and 60 minutes after thrombolysis according to PIS, as an early marker of reperfusion after thrombolytic therapy when added to classical criteria.

## Methods

This was an observational, prospective study. The study was approved by the local ethics committee and all patients or their legal representatives signed an informed consent form before participating in the study.

### Patients

We selected patients with STEMI that sought medical care at public health centers in the city of Sao Paulo, Brazil, who had undergone thrombolytic therapy using TNK, referred for angiography in a tertiary hospital, regardless of electrocardiographic criterion for reperfusion (ST-segment resolution > 50%). Only patients with primary diagnostic of myocardial infarction, considered eligible to thrombolytic therapy in PIS were consecutively included. One hundred and ten patients were initially included, and six were excluded for electrocardiographic reasons. Exclusion criteria were: known contraindications to fibrinolysis, and electrocardiographic findings that could affect QT interval measurements, such as bundle branch block, atrial fibrillation or previous myocardial infarction.

Thus, in the present study, 104 patients of both sexes were included, all of them with primary AMI, treated with TNK within 6 hours of symptoms’ onset at primary care centers and subsequently referred for coronary angiography at a tertiary hospital within 2-24 hours of fibrinolysis, or immediately, in situations when a rescue therapy was needed. The operation of the STEMI network in Sao Paulo has been previously published.^7^^,^^8^

Clinical and demographic data of the patients were obtained. An experienced echocardiographer, who did not know about their clinical history performed the measurements of the ventricular ejection fraction on the fifth day following AMI in all patients.

### Electrocardiographic analysis

Electrocardiographic analysis consisted of an ECG before and 60 minutes after fibrinolysis, using certified and calibrated devices, with patients in prone position. Two independent observers, unaware of patients’ clinical characteristics, analyzed the ECG results. The criteria to undergo electrocardiographic reperfusion was a reduction in ST-segment greater than 50% in the highest lead within 60 minutes of fibrinolytic administration.

QT interval was manually measured using a digital caliper, with a lineal, non-contact measurement system, with a resolution of 0.1 mm/0.01’’, accuracy of ± 0,2 mm / 0.001" (< 100 mm) and ± 0.03 mm / 0.01"(>100 - 200 mm) and repeatability of 0.1 mm / 0.01". QT values were converted to milliseconds (ms) and corrected for heart rate by the Hodges’ linear method using the formula [QTc = QT + 1.75 (RR - 60)]. In order to minimize intraobserver variability, QT interval was calculated by the mean of three measurements in consecutive QRS complexes and in all ECG leads. Kappa coefficient was calculated to minimize the interobserver variability. QT measurements were performed using the tangent method, in which the end of T-wave was defined as the intersection of this tangent with the baseline, at the maximal slope at the end of the QT interval.^16^ In the presence of a U-wave, the end of T wave was taken as the nadir between T and U waves. Additionally, we excluded from the analysis all ECG leads where some variables, particularly the T-wave, could not be clearly determined.

QTcD was defined as the difference between the maximum (QT_max_) and minimum QT (QT_min_) interval in 12-lead ECG. Regional dispersion was calculated as the difference between QT_max_ and QT_min_ only in leads with ST-segment elevation. Acute anterior wall myocardial infarction was defined as ST-segment elevation in DI, aVL, V1-V3 or V1-V6 leads, whereas non-anterior wall myocardial infarction defined as ST-segment elevation in DII, DIII, aVF, and V_5_-V_6_ leads.

### Angiographic analysis

Angiographic analysis was performed in a tertiary hospital according to a PIS protocol previously described. Two experienced hemodynamic technicians (more than 15 years of practice), unaware of any information that could affect angiographic analysis, analyzed the epicardial flow according to TIMI flow grade,^17^ and myocardial perfusion according to myocardial Blush grade.^18^ Myocardial blush, defined as contrast density in myocardial microcirculation (Chart 1), was assessed only in patients with TIMI3 grade.

**Chart 1:** Definitions for myocardial perfusion (microperfusion) by Myocardial Blush Grade

**Grade 0 (absence of myocardial perfusion):** absence of myocardial blush or contrast density
**Grade 1 (minimal myocardial perfusion):** minimal myocardial blush or contrast density
**Grade 2 (partial myocardial perfusion):** moderate myocardial blush or contrast density, but less than that obtained during contrast injection into a contra-lateral or ipsilateral non-infarcted-related coronary artery
**Grade 3 (complete myocardial perfusion):** normal myocardial blush or contrast density, comparable with that obtained during contrast injection into a contra-lateral or ipsilateral non-infarcted-related coronary artery

Adapted from Van 't Hof et al.^18^

### Statistical analysis

Numerical data were expressed as mean and standard deviation (SD) in case of variables with normal distribution, or as median and interquartile range (IQR) in case of quantitative variables with non-normal distribution. The normality of data distribution was tested with the Shapiro-Wilk test and the Kolmogorov-Smirnov test; kurtosis and asymmetry of data distribution were also examined. Categorical variables were expressed as number (n) and percentage (%) and compared by the Pearson’s chi-square test, or Fisher’s exact test, as appropriate. Continuous variables were compared by Student’s t-test for independent samples or the Mann-Whitney test, as appropriate. Within-group comparisons were made by t-test for related samples or the Wilcoxon test. All tests were two-tailed, and a p-value < 0.05 was considered statistically significant. Area under the ROC (receiver operating characteristic) curves, based on C-statistics, were constructed to determine optimal cut-off values for some of the variables. All tests were performed using the Statistical Package for Social Sciences (SPSS)^®^ software version 17.0, da SPSS Inc, Chicago, IL, USA.

## Results

### Baseline characteristics of the population

A total of 104 patients attending public primary care centers, with clinical and electrocardiographic diagnosis of STEMI, treated with a fibrinolytic agent (TNK) and submitted to coronary angiography within 2-24 hours thereafter were included in the study. Main demographic and clinical characteristics of these patients are described in Table 1. Age ranged from 35 to 74 years old, and most patients were men. Time (median and IQR) between symptom onset and initiation of thrombolytic therapy was 180 minutes (120-240 minutes).

**Table 1:** Baseline clinical and epidemiological characteristics of the patients (n = 104)

Age (years)	Men n (%)	Type 2 DM n (%)	SAH n (%)	Dyslipidemia n (%)	Smokers n (%)	Symptom onset (min), m ± SD
55.6 ± 8.78	66 (62.9)	21 (20)	60 (57.1)	36 (34.3)	51 (48.6)	192.16 ± 94.35

Data expressed as mean and standard deviation (m±SD), or number and percentage, n (%), DM: diabetes mellitus; SAH: systemic arterial hypertension

### Localization of infarction

AMI was classified according to the ventricular wall involved. For statistical analysis purpose, AMI was grouped into anterior (n = 42) and non-anterior wall infarction (n = 62).

### Distribution of QTc and QTcD by electrocardiographic criterion for reperfusion

Sixty-seven (64%) patients met the electrocardiographic criterion for reperfusion. Electrocardiographic tracings were analyzed by two independent observers, with a Kappa coefficient of 0.84. Patients were categorized into two groups - patients with signs of reperfusion and patients without signs of reperfusion, considering only a ST-segment resolution of 50% or more. Values of QTc and QTcD before and after fibrinolysis are shown in Table 2. QTc and QTcD intervals in all 12 leads were not different between the groups. Regional QTcD increased in patients with criterion for reperfusion and, considering the involvement of ventricular wall, in patients with anterior wall myocardial infarction with criterion for reperfusion (p = 0.023) (Table 3).

**Table 2:** QT interval corrected for heart rate (QTc) and QTc dispersion behavior in the 12 leads and in the leads with ST-segment elevation only (regional QTcD) in patients who met and in those who did not meet electrocardiographic criteria for reperfusion

Variable	With ST-segment resolution (n =67)	p-value	Without ST-segment resolution (n =37)	p-value
QTc (ms), m ± SD	**Pre-TNK**	0.25	**Pre-TNK**	0.06
	423.79 ± 27.89		416.10 ± 26.17	
	**Post-TNK**		**Post-TNK**	
	429.02 ± 44.60		424.86 ± 24.12	
QTcD (ms), md (IQR)	**Pre-TNK**	0.28	**Pre-TNK**	0.29
	59.0 (45-84)		61 (42-73.5)	
	**Post-TNK**		**Post-TNK**	
	63.0 (47-76)		64 (44.5-90)	
Regional QTc (ms), ± SD	**Pre-TNK**	0.12	**Pre-TNK**	0.24
	420.30 ± 26.27		420.00 ± 30.67	
	**Post-TNK**		**Post-TNK**	
	430.00 ± 45.70		423.89 ± 31.95	
Regional QTcD (ms), md (IQR)	**Pre-TNK**	0.01	**Pre-TNK**	0.13
	28 (16-44)		11.5 (23-44)	
	**Post-TNK**		**Post-TNK**	
	33 ± (20-59)		42 (20-64)	

Data expressed as mean and standard deviation (m ± SD); median and interquartile range, md (IQR); QTcD: QTc dispersion; regional QTc: mean QTc in infarcted area (leads with ST-segment elevation); regional QTcD: regional QTc dispersion (leads with ST-segment elevation); TNK: tenecteplase. Student's t-test for related samples or Wilcoxon test, as appropriate.

**Table 3:** Regional QT interval, corrected for heart rate (QTc) in anterior wall infarction in patients with or without ST-segment resolution and patients with or without TIMI 3 and Blush 3 (n = 42)

Variable	With ST-segment resolution n = 23	p-value	Without ST-segment resolution (n = 19)	p-value
Regional QTc (ms), m ± SD	**Pre-TNK**	0.35	**Pre-TNK**	0.17
	428.54 ± 28.24		419.56 ± 28.44	
	**Post-TNK**		**Post-TNK**	
	429.75 ± 42.59		424.26 ± 30.55	
Regional QTcD (ms), md (IQR)	**Pre-TNK**	0.023	**Pre-TNK**	0.07
	28 (17.5-51.25)		21.5 (9.5-39.25)	
	**Post-TNK**		**Post-TNK**	
	40 (30-66.7)		38.5 (17.5-59)	
	T3B3 (+) n =18	p-value	T3B3 (-) n =24	p-value
Regional QTc (ms), m ± SD	**Pre-TNK**	0.26	**Pre-TNK**	0.70
	425.53 ± 28.24		421.66 ± 28.44	
	**Post-TNK**		**Post-TNK**	
	439.88 ± 42.59		417.62 ± 30.55	
Regional QTcD (ms), md (IQR)	**Pre-TNK**	0.006	**Pre-TNK**	0.07
	23 (15.75-39.25)		25 (18-46)	
	**Post-TNK**		**Post-TNK**	
	38 (24.25-73.0)		42 (21-61)	

Data expressed as mean and standard deviation (m±SD); median and interquartile range, md (IQR); regional QTc: regional QTc in anterior wall infarction; regional QTcD: regional dispersion of the QTc interval in anterior wall infarction; TNK: tenecteplase; T3B3 (+): TIMI 3 and Blush grade 3; T3B3 (-): TIMI < 3 and Blush < 3. Student's t-test for related samples, or Wilcoxon test, as appropriate.

### Distribution of QTcD by angiographic data

Patients were categorized into two groups according to TIMI and Blush grades. Patients with optimal reperfusion, *i.e*., TIMI 3 and Blush grade 3 - group T3B3 (+) - and those with TIMI < 3 and Blush < 3 - group T3B3 (-). Regional QTcD for anterior wall infarction significantly increased in the T3B3(+) group (p = 0.06), but not in non-anterior wall infarction (p = 0.77). To rule out the possibility of measurement bias in non-anterior wall infarction, regional QTcD in unipolar leads (V_1_-V_6_) was compared with that in bipolar leads, with no statistically significant difference.

### Distribution of coronary flow by TIMI and Blush grades

**Figure 1:**
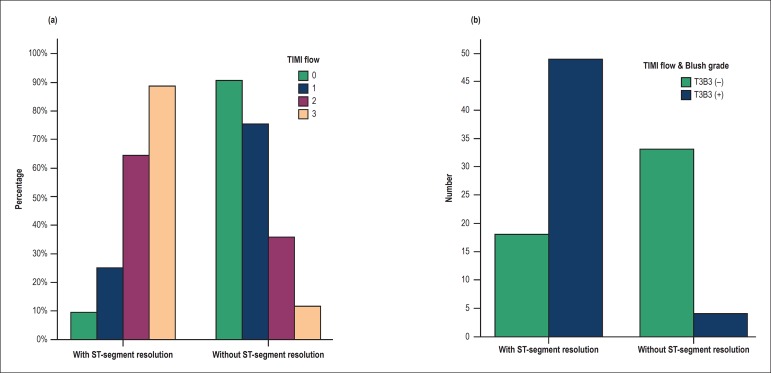
Distribution of patients by the presence of ST-segment resolution (classical electrocardiographic criteria for reperfusion) and angiographic profile of TIMI flow (1a) or perfusion pattern (TIMI flow and Bulsh grade); in the culprit artery; T3B3 (+): patients with TIMI 3 and Blush grade 3 in the culprit artery; T3B3 (-): patients with TIMI 3 and Blush grade < 3 in the culprit artery (1b).

Distribution of the flow in the culprit artery according to TIMI grade flow 0, 1, 2 and 3 was 20.2%, 7.7%, 13.5% and 58.7%, respectively. Figure 1 depicts (a) percentage distribution of patients according to TIMI grade flow and the electrocardiographic criterion for reperfusion (ST-segment resolution); (b) distribution of patients (in relative frequency) according to TIMI and Blush scores (T3B3) and ST-segment resolution. Few patients with TIMI3 did not show adequate myocardial perfusion according to myocardial Blush grade. Distribution of myocardial blush grades 0, 1, 2 and 3 was 4.9%, 3.3%, 4.9% and 86.9%, respectively in patients with TIMI 3.

**Figure 2:**
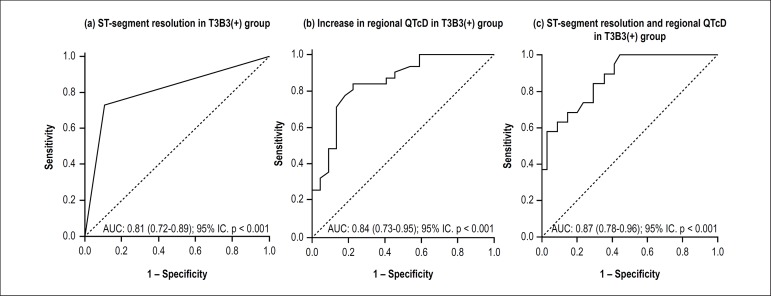
ROC curves for the classical electrocardiographic criterion for reperfusion (ST-segment resolution); regional dispersion of the QT interval, corrected for heart rate (QTc); and ST-segment resolution combined with regional dispersion of the QTc interval in patients with optimal reperfusion profile, i.e., TIMI flow and Blush grades 3 [T3B3 (+)]. (a) In patients with ST-resolution, the area under the ROC curve was 0.81 [(0.72-0.89); 95%CI, p < 0.001) to detect TIMI flow 3 and Blush 3 [T3B3(+)]; (b) increased regional QTc dispersion 60 minutes after thrombolysis resulted in an area under the ROC curve of 0.84 [(0.73-0.95); 95%CI, p < 0.001 to detect T3B3 (+), using a cutoff point of > 13 ms, a 94% sensitivity and a 74% specificity were obtained; (c) increased regional QTcD associated with ST-segment resolution 60 minutes after thrombolysis resulted in an area under the ROC curve of 0.87 [(0.78-0.96); 95%CI, p < 0.001 to detect T3B3 (+). Using a cutoff point of > 13 ms, a 93% sensitivity and a 71% specificity were obtained. Six patients (approximately 6%) could be reclassified based only on electrocardiographic measurements. Validated by angiographic criteria of coronary reperfusion in this cohort of patients treated with pharmaco-invasive strategy.

With respect to the prediction of optimal coronary reperfusion [T3B3(+)], the criterion for reperfusion by ECG and analysis of QTcD showed a positive predictive value of 73%, negative predictive value of 89%, sensitivity of 93% and specificity of 73%. Baseline demographic characteristics according to TIMI/Blush were not different between T3B3(+) and T3B3(-), except for left ventricular ejection fraction, which was lower in T3B3(-) [(52.6 ± 9.8 *vs* 47.8 ± 8.5; p =0.009)] (Table 4). ECG parameters were not different before and 60 minutes after thrombolysis (Table 5). ROC curves were constructed to evaluate the classification performance of the regional QTcD and to establish the best cutoff point, as illustrated in Figure 2.

**Table 4:** Clinical characteristics in the groups of patients with or without angiographic criteria for adequate reperfusion according to TIMI flow and Blush grades

Characteristics	T3B3 (+)	T3B3 (-)	p-value
	n =53	n =51	
Age (years), md (IQR)	54 (47-63)	56 (52-62)	0.51
Male, n (%)	28 (52.8)	38 (74.5)	0.02
Type 2 DM, n (%)	8 (15.1)	13 (25.5)	0.19
Hypertension, n (%)	27 (50.9)	33 (64.7)	0.16
Dyslipidemia, n (%)	15 (28.3)	21 (41.2)	0.17
Smokers, n (%)	24 (45.3)	27 (53)	0.43
Time for TNK administration, (min): md -IQR	185 (137-257)	138 (110-240)	0.18
Time < 180, (min): n (%)	32 (60)	22 (43)	0.12
Ejection fraction, (%): m ± DP	52.6 ± 9.8	47.8 ± 8.5	0.009
Anterior AMI, n (%)	18 (34)	24 (47)	0.17
Non-anterior, n (%)	35 (66)	27 (53)	0.17

Data expressed as mean and standard deviation (m ± SD), median and interquartile range (md, IQR), number and percentage, n (%); T3B3 (+): patients with TIMI 3 and Blush grade 3 in the culprit artery; T3B3 (-): patients with TIMI 3 and Blush grade < 3 in the culprit artery; DM: diabetes mellitus; AMI: acute myocardial infarction; TNK: tenecteplase. Categorical variables were compared by Pearson's chi-square test or Fisher's exact test, and continuous numerical variables were compared by the Student's t test for independent sample or Mann-Whitney test, as appropriate.

**Table 5:** Electrocardiographic parameters evaluated before and after tenecteplase (TNK) administration in patient with TIMI 3 and Blush grade 3 [T3B3 (+)] and patients with TIMI < 3 and Blush grade < 3 [T3B3 (-)] in the culprit artery

**Pre-TNK**	**T3B3 (+)**	**T3B3 (-)**	**p-value**
**N**	**53**	**51**	
QTc (ms), m ± SD	421.56 ± 28.51	423.29 ± 25.77	0.72
QTcD (ms), md (IIQ)	59 (44-82)	59 (43-81)	0.97
Regional QTc (ms), m ± SD	418.86 ± 27.01	423.55 ± 30.41	0.38
Regional QTc (ms), md (IQR)	25 (11.5-40)	29 (18-50)	0.09
**Pre -TNK**	**T3B3 (+)**	**T3B3 (-)**	**p-value**
**N**	**18**	**24**	
Regional QTcD (ms), md (IIQ)	23 (11.75-39.25)	25 (18-46)	0.65
**Post -TNK**	**T3B3 (+)**	**T3B3 (-)**	**p-value**
**N**	**53**	**51**	
QTc (ms), m ± DP	426.90 ± 43.98	431.94 ± 27.47	0.42
QTcD (ms), md (IIQ)	62 (49-75)	66 (40-91)	0.62
Regional QTc (ms), m ± DP	430.53 ± 44.01	424.14 ± 36.12	0.19
Regional QTcD (ms), md (IIQ)	33 (20-59)	42 (19-63)	0.71
**Post -TNK**	**T3B3 (+)**	**T3B3 (-)**	**p-value**
**N**	**18**	**24**	
Regional QTcD (ms), md (IIQ)	38 (24.25-73)	42 (21-61)	0.05

Data expressed as mean and standard deviation (m ± SD), median and interquartile range (md and IQR). QTc: mean QT interval, corrected for heart rate in the 12 leads; QTcD: dispersion of the QTc interval in the 12 leads; regional QTc: mean regional QTc in anterior wall infarction; regional QTcD: regional QTc dispersion in anterior wall infarction. Continuous numerical variables were compared by the Student's t-test for independent samples or the Mann-Whitney test, as appropriate.

**Figure 3:**
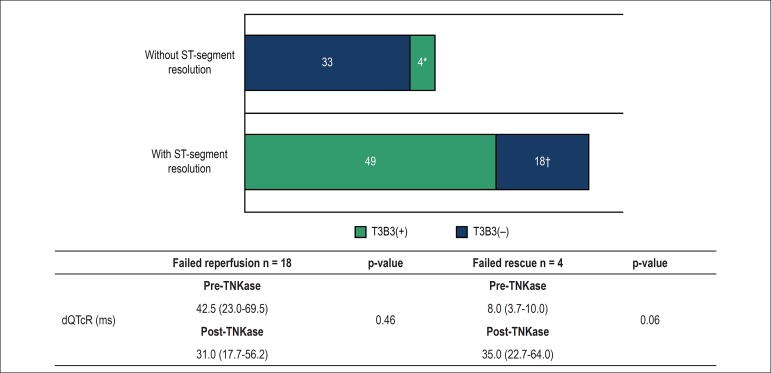
Behavior of the regional QT interval, corrected for heart rate, in subgroups of patients with failed reperfusion or failed rescue; data expressed as median and interquartile range (m,IQR); QTcD: dispersion of the QTc interval; TNK: tenecteplase; T3B3 (+): patients with TIMI 3 and Blush grade 3 in the culprit artery; T3B3 (-): patients with TIMI 3 and Blush grade < 3 in the culprit artery; Wilcoxon test. *Failed rescue: patients without ST-segment resolution and with optimal coronary and tissue perfusion [T3B3 (+)]; †failed reperfusion: patients with ST-segment resolution, without optimal coronary and tissue perfusion [T3B3 (-)].

If we consider only patients in which the classical electrocardiographic criterion for reperfusion failed to identify coronary reperfusion, there were 18 patients with ST-segment resolution in which optimal angiographic reperfusion was not achieved (failed reperfusion by ECG), and four patients without ST-segment resolution showed TIMI grade 3 and Blush grade 3 (failed rescue). In the groups with failed reperfusion by ECG, no difference was found in regional QTcD between pre-thrombolysis and post-thrombolysis electrocardiographic analysis (p = 0.46) (Figure 3). Therefore, of the 104 patients who received TNK, we detected incorrect reperfusion in 22 cases (21%).

## Discussion

QT interval between different ECG leads and this range of intervals is considered an index of spatial dispersion of ventricular recovery, serving as a signal of repolarization heterogeneity.^19^ Many studies have shown that patients with increased QTcD (approximately > 60 ms) had 2-3.4 increased risk of cardiovascular mortality. Multivariate analysis of these studies showed a 34% increased cardiovascular risk for each increment of 17ms in QTbD or QTcD > 60 ms in patients with diabetes mellitus without previous AMI.^20^^-^^22^

There is a QTcD variation during the first days of AMI; it increases in the first hours and decreases some days thereafter, especially following fibrinolytic therapy^23^^-^^25^ or revascularization procedure.^26^^,^^27^ A reduction in QTcD in the days following fibrinolysis shows the efficacy of the therapy.^28^ Based on the speculation that changes in QTcD could predict reperfusion assessed 90 minutes after fibrinolysis, in a small study with 47 patients, the authors analyzed QTcD only in precordial leads and found a higher QTcD in the group that met the electrocardiographic criterion for reperfusion. However, the parameter was not predictive of angiographic reperfusion.^29^ One limitation of this study was the small number of patients and the analysis of QTcD in precordial leads only. Another study involving 36 patients did not show any difference in QTcD in the group with criterion for reperfusion on the first day of AMI. Interestingly, the authors observed a decrease in QTcD since the second day of thrombolysis, particularly in the group with anterior wall myocardial infarction.^30^ Another study also reported decreased QTcD six months after AMI.^31^

Our findings indicate an increase in QTcD on ECG 60 minutes after fibrinolysis in patients with angiographic findings of complete vascular and tissue revascularization (TIMI flow and Blush grades 3), especially in anterior wall infarction. On the other hand, different from previous reports on streptokinase and alteplase,^31^^,^^32^ we used TNK, a fibrin-specific, recombinant tissue plasminogen activator, which has been shown better results regarding coronary reperfusion. Besides, we included a larger number of patients compared with previous studies. Regional QTcD in anterior wall infarction significantly increased in ECG obtained 60 minutes after thrombolysis in patients with adequate reperfusion (TIMI 3 and Blush grade 3), reinforcing the idea that QTcD following AMI depends on permeability of the culprit artery, as well on dimension and localization of the ventricular wall involved.

One possible mechanism for our results is based on the effect of cardiac stunning caused by reperfusion injury. Besides, there is evidence that vascular, metabolic, mitochondrial, neuronal, thermal and electric processes contribute to post-reperfusion dysfunction.^33^^-^^35^ Nevertheless, the exact mechanism, the adequate prevention of the ischemia-reperfusion lesion, and above all, the correlation of reperfusion injury with electrocardiographic findings have not been elucidated in the literature.^36^

Considering the correlation between ECG leads and the infarcted area, it is possible to analyze the repolarization of the injured area. Calculation of the regional QTcD estimates heterogeneity of ventricular repolarization in the area at risk. Thereby, the need for a decision-making tool for fibrinolytic therapy emphasizes the importance of post-thrombolysis electrocardiographic reperfusion markers. ECG plays a crucial and more important role in PIS than in primary PCI. The identification of patients that meet reperfusion criteria and of those who should be referred for rescue PCI should be promptly and fast performed. A crucial point is the cost-benefit of the system and the delay in the ideal time between fibrinolysis and PCI. Despite the large variation in this time window in the clinical trials, a time interval of 2-24 hours after successful fibrinolysis.^37^

The classical electrocardiographic criterion for reperfusion has a sensitivity and specificity of 60% and 80%, respectively.^38^ We showed that both sensitivity and specificity increased when regional QTcD was added to ST-segment resolution, suggesting that this method may help to stratify patients in a more accurate way.

Analysis of subgroups did not show significant differences in regional QTcD between patients with at least 50% ST-segment resolution and inadequate flow by angiography [T3B3(-)], *i.e*., patients with failed reperfusion, and patients without ST-segment resolution but who showed angiographic reperfusion [T3B3 (+)].

Our study showed an increase in QTcD and regional QTcD in anterior wall infarction particularly in patients T3B3(+). In agreement with a previous study,^39^ QTcD depends on the localization of AMI, and higher QTcD was observed in the anterior wall as compared with inferior wall acute myocardial infarction. The large area of infarction in this subgroup should have greater influence on repolarization vectors than on non-anterior wall infarction.

This study indicates a possible step forward in the analysis of electrocardiographic variables, in light of current controversies of angiographic data, T3B3(-) showed worse ejection fraction and higher QTcD compared with the T3B3(+) subgroup, which may also have prognostic implications.

Although QTcD is still a matter of controversy in electrocardiology,^40^ some questions remain unanswered in the specialized literature. Studies on electrocardiographic variables using better estimation methods may yield interesting information in many medical scenarios.

### Importance and limitations

So far, there are no studies specifically examining the behavior of regional QTcD in AMI patients who underwent PIS. Therefore, our data need to be further validated and replicated in future studies. Our cohort was relatively small, although larger than in previous studies. Also, advances in the methods used for the measurement of QT interval and ventricular repolarization are still needed. The lack of standardization and systematization negatively affects the accuracy in the measurement of ST-segment and T-wave in the presence of ischemia. Finally, analysis of QTcD by ECG at late follow-up could give interesting information on QTcD behavior.

## Conclusions

Our study suggests that an increase in regional QTcD may detect adequate reperfusion 60 minutes after fibrinolysis, which could be a potential non-invasive method for evaluation of regional perfusion especially in anterior wall infarction.
